# Synthesis of MOF525/PEDOT Composites as Microelectrodes for Electrochemical Sensing of Dopamine

**DOI:** 10.3390/polym12091976

**Published:** 2020-08-31

**Authors:** Season S. Chen, Po-Chun Han, Wai-Kei Kuok, Jian-Yu Lu, Yesong Gu, Tansir Ahamad, Saad M. Alshehri, Hailemichael Ayalew, Hsiao-hua Yu, Kevin C.-W. Wu

**Affiliations:** 1 Department of Chemical Engineering, National Taiwan University, No. 1, Sec. 4, Roosevelt Road, Taipei 10617, Taiwan; season.chen@connect.polyu.hk (S.S.C.); r05524093@ntu.edu.tw (W.-K.K.); 2 Program of Green Materials and Precision Devices, National Taiwan University, Taipei 10617, Taiwan; a242520002000@gmail.com; 3 Department of Chemical and Materials Engineering, Tunghai University, No. 1727, Sec. 4, Taiwan Boulevard, Xitun District, Taichung City 407224, Taiwan; s06310152@go.thu.edu.tw (J.-Y.L.); yegu@thu.edu.tw (Y.G.); 4 Department of Chemistry, College of Science, King Saud University, P.O. Box 2455, Riyadh 11451, Saudi Arabia; tahamed@ksu.edu.sa (T.A.); alshehri@ksu.edu.sa (S.M.A.); 5 Smart Organic Materials Laboratory, Institute of Chemistry, Academia Sinica, Taipei 11529, Taiwan; gishenm@gmail.com

**Keywords:** electropolymerization, dopamine detection, poly(3,4-ethylenedioxythiophene) (PEDOT), electrocatalysis, microelectrode, biosensor

## Abstract

Dopamine (DA) is an important neurotransmitter responsible for the functions and activities of multiple systems in human. Electrochemical detection of DA has the advantages of fast analysis and cost-effectiveness, while a regular electrode probe is restricted to laboratory use because the probe size is too large to be suitable for an in vivo or in vitro analysis. In this study, we have developed porphyrin-based metal organic framework (MOF525) and poly(3,4-ethylenedioxythiophene) (PEDOT)-based composites to modify microelectrode for DA detection. Two types of PEDOT monomers with different functional groups were investigated in this study. By varying the monomer ratios, electrolyte concentrations, and electropolymerization temperature, it was found that the PEDOT monomer containing carboxylic group facilitated the formation of regular morphology during the electropolymerization process. The uniform morphology of the PEDOT promoted the electron transmission efficiency in the same direction, while the MOF525 provided a large reactive surface area for electrocatalysis of DA. Thus, the MOF525/PEDOT composite improved the sensitivity-to-noise ratio of DA signaling, where the sensitivity reached 11 nA/μM in a good linear range of 4–100 µM. In addition, porphyrin-based MOF could also increase the selectivity to DA against other common clinical interferences, such as ascorbic acid and uric acid. The as-synthesized microelectrode modified with MOF525/PEDOT in this study exhibited great potential in real time analysis.

## 1. Introduction

Dopamine (DA) is a crucial neurotransmitter of catecholamine family that is produced in adrenal glands, substantia nigra, ventral tegmental area, and hypothalamus [1]. In neuromodulation, the dopaminergic system plays important roles in cognitive function, motor control, and motivation system [1,2]. An unbalanced dopaminergic activity is implicated to several neurodegenerative diseases such as Parkinson’s, attention deficit and hyperactivity disorder, Huntington’s chorea, and schizophrenia [3,4,5,6]. Conventional detection methods of dopamine include chromatography, fluorimetry, chemiluminescence, mass spectrometry, and capillary electrophoresis [7,8,9,10,11]. Although these methods are mature and known for high sensitivity and selectivity, long analysis time and complex equipment setup limit their in vivo or in vitro measurements of the DA concentration due to the transient nature of release and uptake by living cells [12].

The electrochemical method allows real-time and direct measurement of DA concentration, since DA is an electroactive compound and subjected to reversible oxidation to dopamine-o-quinone upon sufficient potential application. Electrochemical sensors can be divided into biosensors and chemical sensors [13]. Biosensors modified with tyrosinase immobilization have been successfully used in dopamine determination in a biological environment in the presence of ascorbic acid and uric acid [14]. Practical applications of such biosensors are limited by their short lifetime and poor reproducibility [15,16,17]. Alternatively, chemical sensors modified with a wide range of materials such as carbon-based materials [18,19,20], ionic liquid composites [21,22], metal/semiconductors [23,24,25], and organometallic polymers [26,27] are able to provide similar detection limits in comparison with biosensors, and enable the detection of DA in the presence of interfering compounds mimicking biological systems.

Metal-organic framework (MOF) is a type of porous coordination polymer that is formed with metal ions/clusters and organic linkers, displaying ultra-high specific surface area (1000–10,000 m^2^/g) [28,29,30]. Coupled with conductive substances, redox-active MOF materials can be used to modify the electrodes to provide abundant redox active sites, which could enhance the sensitivity and selectivity of biosensing. Our laboratory has previously synthesized poly(3,4-ethylenedioxythiophene) nanotubes (PEDOT NTs) coated with porphyrin-based MOF nanocrystals (MOF-525) as porous electrodes for efficient electrochemical biosensing of DA [26]. The MOF-525-PEDOT composites exhibited a linear DA detection ranging from 2 to 270 µM with a detection limit of 0.04 µM, and a high selectivity toward DA in the presence of ascorbic acid and uric acid. It was found that MOF-25 acted as an electrocatalytic surface, while the PEDOT NTs enabled a rapid electron transport from MOF nanocrystals and vice versa, and served as a redox mediator [26,31].

Development of microelectrochemistry further advances electrochemical sensing of dopamine. In a clinical measurement, online monitoring of the changes of dopamine concentration usually requires the implantation of electrode probes, which could result in the substantial tissue damage by inserting regular large-scale probes [13]. As a result, the microenvironment near the probes would change with time, causing diminished extraction efficiency and underestimation of in vivo quantification [32,33]. Miniaturized electrodes could overcome such issues. A microelectrode is generally defined as at least one side less than 100 µm or the geometric area between 3000 and 15,000 µm^2^ [34]. Microelectrodes can lessen the voltage drop caused by ohmic resistance owing to the small current passing through the microelectrodes [13]. In addition, the small active reaction surface of the microelectrode reduces the electric double-layer effect and allows the fast scan cyclic voltammetry (FSCV) method for real-time analysis. The employments of microelectrodes also improve signal-to-noise ratios due to higher rates of mass transfer and faster response time [35,36].

To synthesize effective microelectrodes for DA determination, appropriate modifications on the microelectrode surface are necessary to enhance the sensitivity and selectivity. Electrochemical polymerization was employed in this study to synthesize the PEDOT-based polymer coating with MOF525, where the former acted as an electron mediator and the latter provided a large reactive area. The objectives of this work are to: (i) synthesize effective microelectrode for dopamine detection with electrocatalytic active material (i.e., MOF525) coupled with conductive material (i.e., PEDOT); (ii) investigate the electropolymerization key parameters such as monomer types, monomer ratios, and electropolymerization time on the formation of polymer and effect on the electrocatalysis; and (iii) evaluate the sensitivity and selectivity toward DA determination.

## 2. Materials and Methods

### 2.1. Synthesis of MOF525

In this study, MOF525 was prepared by the hydrothermal method. In brief, 105 mg of zirconium oxychloride (ZrOCl_2_) and 1.35 g of benzoic acid were completely dissolved in 8 mL of dimethylformamide (DMF) and the mixture was placed in an oven at 80 °C for 2 h. After cooling to room temperature, 46.3 mg of tetra (4-carboxyphenyl) porphin was added to the mixture as the organic ligand, and the mixture was ultrasonicated for 20 min until the color of solution changed from black to green. The solution was then put back into the 80 °C oven for a 24 h reaction. After the reaction finished, the bottom layer of the solution turned to purple, indicating that MOF525 was successfully synthesized. The solid product was collected by a high-speed centrifuge (15,000 rpm, 15 min), and washed with DMF and acetone three times, respectively, to remove all unreacted residues. The resultant product was then placed in a vacuum at room temperature, 40 Pa to dry and stored overnight for further use.

### 2.2. Electropolymerization of 3,4-Ethylenedioxythiophene (EDOT)-Based Monomers

Two types of EDOT monomers (i.e., 3,4-ethylenedioxythiophene methanol (EDOT-MeOH) and 4-(((2,3-dihydrothieno [3,4-*b*] [1,4] dioxin-2-yl) methoxy) methyl) benzoate (EDOT-Ph-COOH)) will be used for polymerization in this study. Under a nitrogen atmosphere, 3 g of EDOT-MeOH, 4.24 g of ethyl 4-(bromomethyl) benzoate, 0.52 g of sodium iodide, and 0.98 g of sodium hydride were added to 25 mL of tetrahydrofuran (THF) to synthesize ethyl 4-(((2,3-dihydrothieno [3,4-b] [1,4] dioxin-2-yl) methoxy) methyl) benzoate (EDOT-Ph-COOEt) intermediate. After stirring until fully dissolved, the mixture was placed in an ice bath, where 10 mL of 1.4 M ethyl 4-(bromomethyl)benzoate in THF was slowly added. The ice bath was then removed, and the mixture was stirred at room temperature for one day. After the reaction, 40 mL of deionized water was added to the mixture, followed by addition of 60 mL of ethyl acetate for extraction. The organic phase was retained and repeated the extraction for two times. After that, 100 mL of deionized water was added to the extracted organic phase to ensure that most ions and water-soluble impurities were removed. Anhydrous MgSO_4_ powder was added to remove water. After filtration and removing most of the solvent by rotary evaporation, the product was further purified by column chromatography, where the stationary phase was silica gel and the mobile phase was 8 vol % ethyl acetate in hexane. At last, 4.38 g of colorless product was obtained, and the yield of EDOT-Ph-COOEt was 75%.

To obtain EDOT-Ph-COOH, the EDOT-Ph-COOEt was hydrolyzed in an alkaline environment. Under nitrogen atmosphere, 2.48 g of EDOT-Ph-COOEt was added to 20 mL THF with continuous stirring. After fully dissolution, 15 mL of 2 M NaOH solution was added to the solution and the mixture was heated at 90 °C for 24 h under stirring. After the reaction, 1 N of HCl was used to adjust the pH of the solution below 3, followed by the addition of 60 mL of ethyl acetate for extraction. The organic phase was retained, and the extraction processes were repeated twice. The purification processes were similar to that for EDOT-Ph-COOEt with MgSO_4_ powder and column chromatography.

The monomers of EDOT-MeOH and EDOT-Ph-COOH were polymerized into poly (EDOT-MeOH; referred to as PEM) and poly (EDOT-ph-COOH; referred to as PEpC), respectively, by electrochemical oxidative polymerization. The Pt/Ir microelectrode was used as the working electrode, Ag/Ag^+^ as the reference electrode, and the platinum wire was the counter electrode. The microelectrode was first cleaned under ultrasound in 0.1 M NaOH solution for 10 min, and then immerse in piranha solution for 10 s to remove organic impurities attached to the electrode. The electrode was further ultrasonicated in deionized water, acetone, isopropanol and ethanol for 10 min each, and then dried and stored under vacuum. Then, a voltage of 1.4 V was applied to the dichloromethane (DCM) solution containing 1.5 mL of 10 mM monomer and 0.1 M tetrabutylammonium perchlorate (TBAP) in an ice bath for oxidative electropolymerization for 100 s. After the reaction, the microelectrode was cleaned by pure DCM for use.

### 2.3. Preparation of MOF525/PEDOT Composites Modified Microelectrode

The MOF525 was added to DMF to obtain a concentration of 8 g/L, then PEDOT-based PEM and PEpC microelectrodes synthesized in the previous step were immersed into the MOF525 solution. The mixture was placed in a 0 °C refrigerator for 24 h to allow fully adsorption of MOF525 onto PEDOT surface. After reaction, the microelectrodes were removed from MOF525 solution and immersed in acetone for anther 30 min to remove DMF solution on the surface. The resultant MOF525/PEDOT microelectrodes were dried under vacuum and stored for further use.

### 2.4. Material Characterizations

X-ray diffraction (XRD) analyzer was used to analyze the diffraction pattern of MOF525 from a scanning angle of 3–40° in order to evaluate the product purity The as-synthesized MOF525 was analyzed by the nitrogen adsorption–desorption isotherm (Micromeritics ASAP2020) to determine the Brunauer–Emmett–Teller (BET) specific surface area, and the pore size distribution was calculated by the density functional theory method. To verify the monomer structure synthesized in this study, nuclear magnetic resonance spectroscopy (NMR, Bruker AV-400) was used. Prior to the analysis, the appropriate amount of the monomer was dissolved in the deuterated chloroform. The surfaces of microelectrodes before and after modification were recorded by field emission scanning electron microscope (FE-SEM, Nova NanoSEM 230), and the energy dispersive X-ray spectrometer (EDX) was used to analyze the elemental distribution on the electrodes.

### 2.5. Electrochemical Determination of Dopamine

The performance of the microelectrodes was evaluated using a three-electrode system with the CHI900 electrochemical workstation. The modified/unmodified microelectrode was used as the working electrode, Ag/AgCl^+^ as the reference electrode, and the platinum wire was the counter electrode. Acetic acid buffer solutions were used to prepare dopamine solutions of various concentrations as the detection solutions. The amount of solutions added to the cell tank and the insertion depth of the electrodes were remained consistent in the tests. Cyclic voltammetry (CV) and differential pulse voltammetry (DPV) methods were used to analyze the characteristics of the electrodes before and after MOF525/PEDOT composite modification.

## 3. Results and Discussion

### 3.1. NMR Analysis of EDOT Monomers

**Figure 1:**
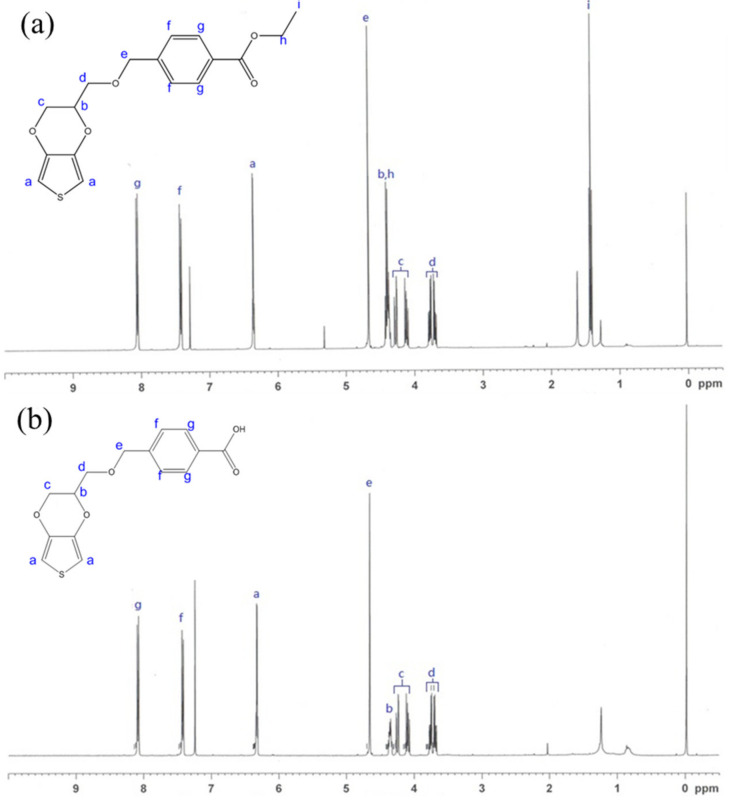
^1^H-NMR spectra of (**a**) EDOT-Ph-COOEt and (**b**) EDOT-Ph-COOH.

The ^1^H-NMR spectrum of the purified EDOT-Ph-COOEt showed chemical shifts at 1.42 (–OCH_2_C**H_3_**), 3.68–3.78 (–CHC**H_2_**O–), 4.09–4.29 (–OC**H_2_**CH–), 4.36–4.43 (– (C**H**)CH_2_O–, –OC**H_2_**CH_3_–), 4.67 (–OC**H_2_**C–), 6.36 (–SC**H**C–), 7.42 (–CH_2_(C)C**H**–), and 8.06 (–C**H**(C)C–), which were corresponding to the characteristic peaks of EDOT. In addition, the characteristic peaks of ^1^H on the benzene ring, ethyl ester, and ethers were also observed (Figure 1a), confirming the successful synthesis of EDOT-Ph-COOEt as the intermediate for EDOT-Ph-COOH. After hydrolysis of EDOT-Ph-COOEt, it was found that the peak at 1.42 ppm disappeared in the ^1^H-NMR spectrum, and the peak intensities between 4.36 and 4.43 ppm were greatly reduced (Figure 1b). These results indicated that EDOT-Ph-COOEt was successfully hydrolyzed to EDOT-Ph-COOH, which would be used as one of the monomers in this study.

### 3.2. Characterization of MOF525

**Figure 2:**
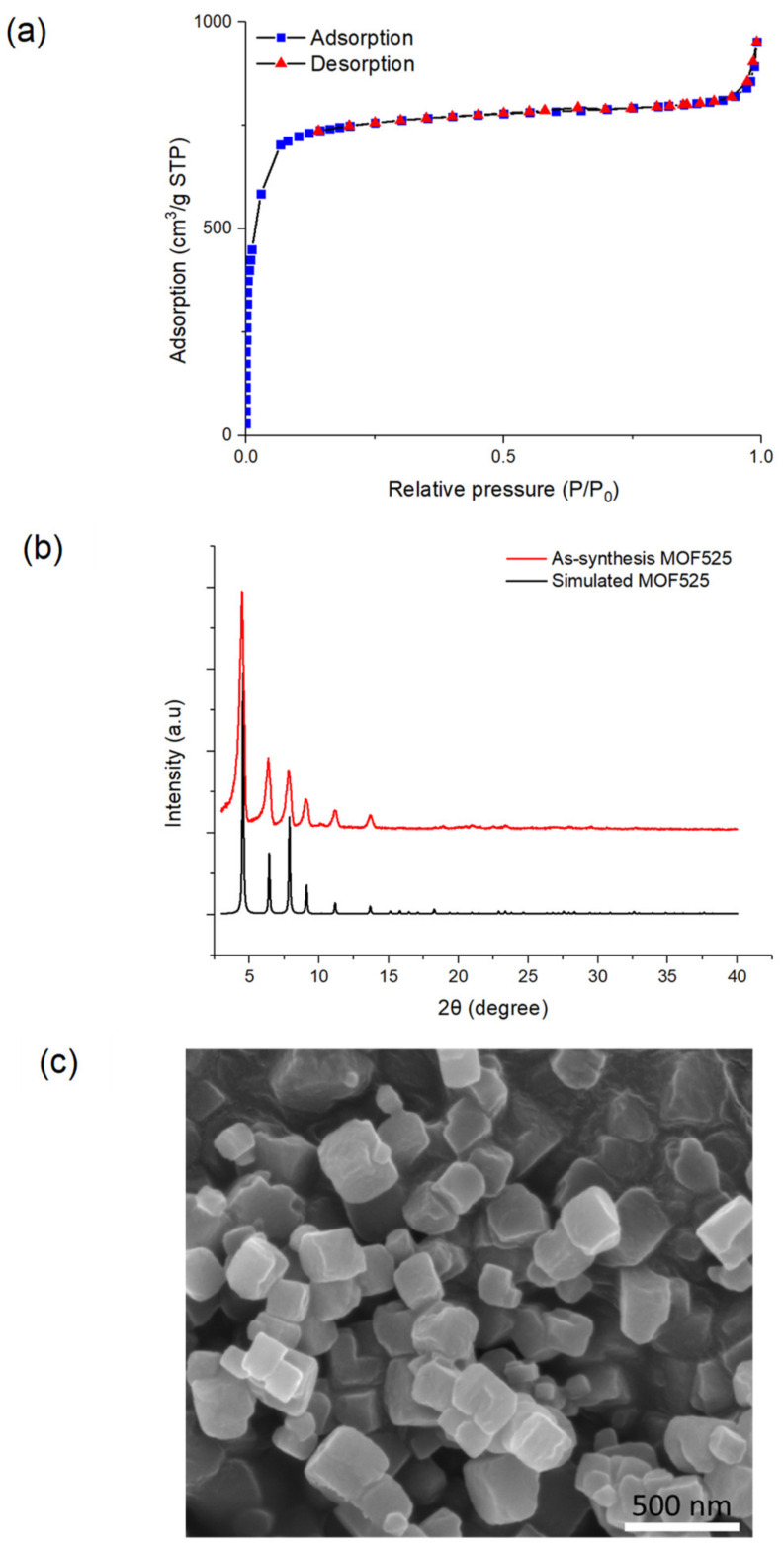
MOF525 characterizations by (**a**) nitrogen adsorption-desorption isotherms, (**b**) XRD, and (**c**) SEM.

Hydrothermal method was used to prepare MOF525 in this study. As shown in Figure 2a, the porphyrin-based MOF525 exhibited a high BET specific surface area of 2580 m^2^/g, which is comparable to previous studies [26,37]. The pore size of MOF525 was calculated as 2.28 nm using the density functional theory method for a cylindrical-pore model. The XRD pattern of MOF525 showed distinct diffraction peaks at 2*θ* = 4.6°, 6.5°, 8.0°, and 9.2° (Figure 2b), indicating high crystallinity of the as-synthesis MOF525, which agreed with the simulation result [38]. The SEM image showed the cubic structure of MOF525 nanocrystals (Figure 2c).

### 3.3. MOF525/PEM Composites Modified Microelectrode

**Figure 3:**
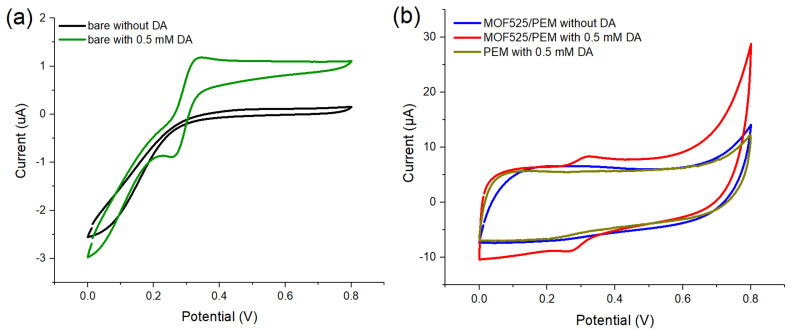
Cyclic voltammetry (CV) traces of (**a**) unmodified microelectrode and (**b**) modified microelectrodes in the presence of 0- or 0.5-mM DA (scan rate: 30 mV/s).

The microelectrode was first modified with MOF525 and PEM (i.e., poly (EDOT-MeOH)) as MOF525/PEM composites, and tested by CV at a scanning rate of 30 mV/s in the presence of acetate buffer solution (ABS, pH 5) containing 0 or 0.5 mM DA. The CV traces of the unmodified microelectrode showed an oxidative peak at 0.34 V after the addition of DA, indicating the unmodified Pt/Ir microelectrode was also capable of electrocatalytic oxidation of DA (Figure 3a). It should be noted that Pt itself was highly catalytic active for H^+^, which will reduce H^+^ to H_2_ at 0.33 V [39,40]. This result implied that the potential window of hydrogen reduction was approximately 0.3–0.8 V. The similar boundaries with the DA oxidation potential caused difficulty in the interpretation of the oxidation peak current of DA. After modification of PEM or MOF525/PEM composites, the potential window shifted to 0–0.6 V (Figure 3b). The PEM modified microelectrode did not show a significant oxidation peak, while the MOF525/PEM modified microelectrode showed a moderate oxidation peak at 0.32 V. These results proved that redox active MOF525 can provide the reactive area for electrocatalysis of DA. However, the background current also increased significantly upon electrode modification caused by the capacitance. The ratio of the oxidation peak to the background current (S/B ratio) was only 1.26. In addition, the background currents compared with PEM and MOF525/PEM modified microelectrodes showed negligible difference (Figure 3b), implying that the increase of background current was mainly from PEM.

**Figure 4:**
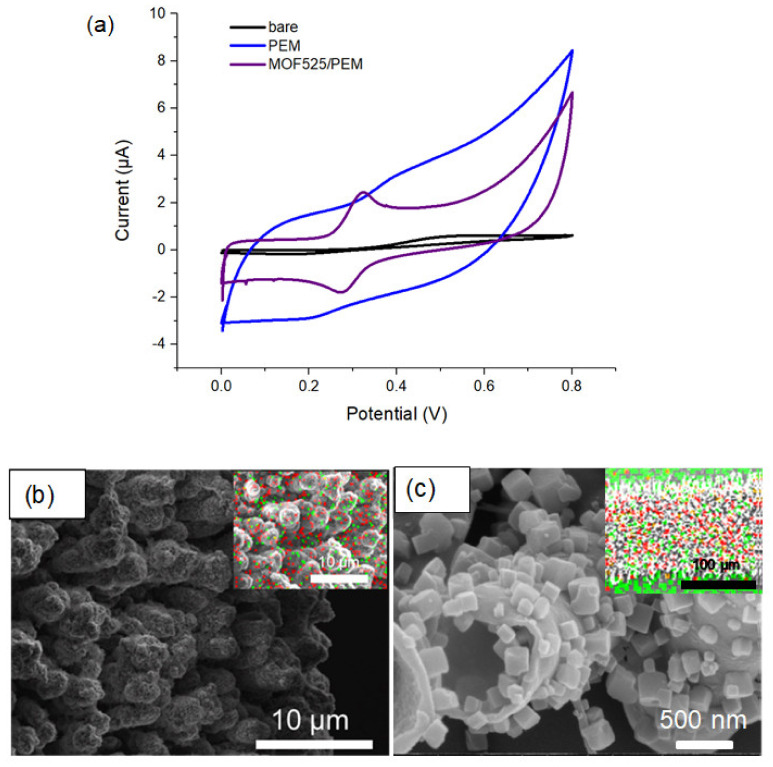
(**a**) CV traces of bare and modified flat microelectrodes; SEM images of MOF525/PEM on the (**b**) Pt/Ir microelectrode and (**c**) flat electrode (inset: EDX of elemental distribution. Red: S (representing PEM), and green: Zr (representing MOF525)).

To further investigate the reason of the changes of background current, the same preparation techniques were used to modify a flat strip microelectrode (approximately 100 µm in width). In the CV test, the oxidation peak on the MOF525/PEM modified flat electrode was found at 0.32 V, similar to the value of modified Pt/Ir microelectrode (Figure 4a). Meanwhile, the S/B ratio was significantly improved to 3.21. The EDX results showed that PEM and MOF525 were successfully incorporated onto both microelectrodes (Figure 4b,c), while the surface morphologies of the modification on two microelectrodes were different. On the Pt/Ir microelectrode, the structure of PEM presented a solid cylinder form (Figure 4b), while the PEM formed as hollow tubes on the flat microelectrode (Figure 4c). For the hollow tube structure of PEM, MOF525 was adsorbed on both the inner layer and outer layer of the tube, which significantly increased the available area for reaction. It is inferred that the electrode surface tension may change with the electrode geometry, a compacted PEM formed during polymerization led to a decrease of available reaction area, which resulted in a weak oxidation peak [41,42].

The main factors affecting the structure of PEDOT-based polymers include: (i) the number of hydrogen bonds; (ii) the temperature of electropolymerization; (iii) the concentration of electrolyte; and (iv) the molecular structure of EDOT functional groups. In the process of electropolymerization, stronger hydrogen bonds between monomer molecules would induce the specific micelles by self-assembling with electrolyte and solvent molecules, which would further lead to a tighter arrangement between polymers [43,44]. Electrolyte ions with non-polar group such as TBAP^+^ can assist the process of self-assembly into micelles [43]. The longer the monomer structure, the easier to form the micelles with a specific shape [44]. In addition to the interaction between molecules in the electropolymerization process, a low temperature can also promote an intermolecular arrangement in a more regular way and then influence the surface tension of the electrode [45]. To enhance the S/B ratio of DA detection, the polymerization conditions were adjusted based on these major factors.

**Figure 5:**
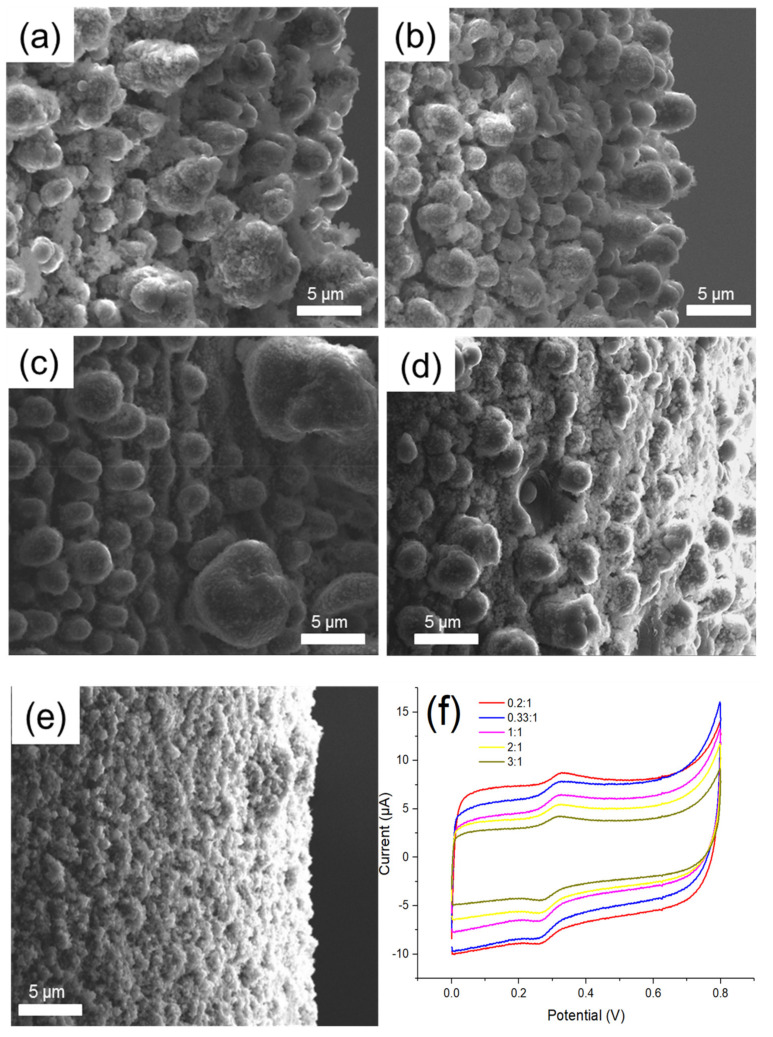
The SEM images of MOF525/PEM composites synthesized with various EDOT-MeOH/EDOT ratios: (**a**) 3:1; (**b**) 2:1; (**c**) 1:1; (**d**) 0.33:1; (**e**) 0.2:1; and (**f**) the CV traces of MOF525/PEM composites modified microelectrode synthesized under various EDOT-MeOH/EDOT ratios.

To control the number of PEM hydrogen bonds, the EDOT monomer without any functional group was used to co-polymerize with EDOT-MeOH. The total monomer concentration was fixed, while the ratios of EDOT-MeOH to EDOT were set as 3:1, 2:1, 1:1, 0.33:1, and 0.2:1. The SEM images revealed that the cylinder morphology of PEM gradually changed to spherical shape and then to the powder form as the EDOT-MeOH content decreased (Figure 5a–e). The morphological changes also corresponded with the variations shown in CV traces. As the content of EDOT-MeOH reduced, the background current increased (Figure 5f), resulting in a decreasing S/B ratio (Table 1). These results implied that reducing EDOT-MeOH ratio led to insufficient hydrogen bonds to maintain the cylinder structure of the polymer. The loss of three-dimensional structure also reduced reactive area and hence lowered the S/B ratio. Thereby, regulating the number of hydrogen bond was not a feasible approach to synthesize the ideal polymer structure in this study.

**Figure 6:**
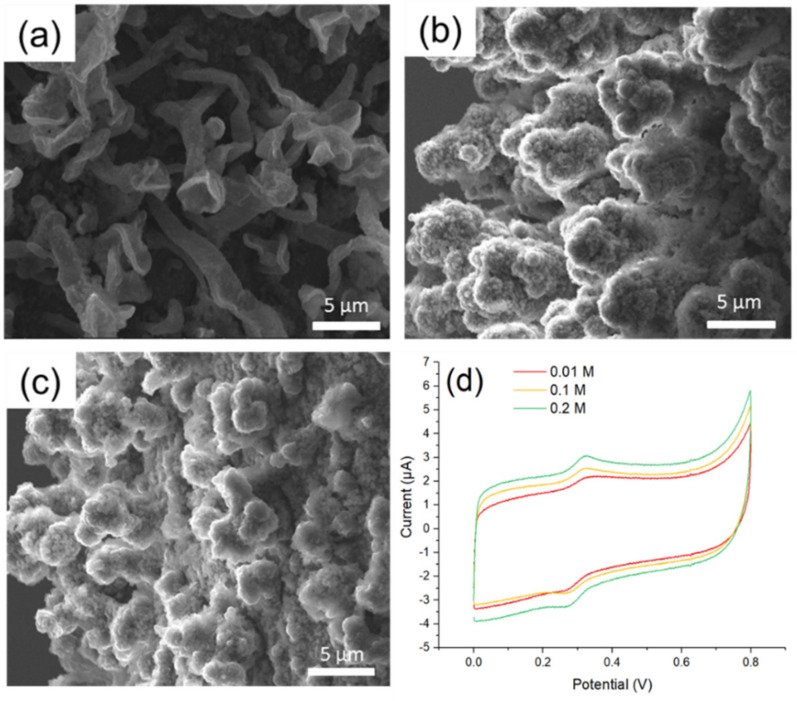
The SEM images of MOF525/PEM composites synthesized under: (**a**) 0.01 M; (**b**) 0.1 M; and (**c**) 0.2 M TBAP solution; and (**d**) the CV traces of MOF525/PEM composites modified microelectrode synthesized under various TBAP concentrations.

In the next step, the concentration of supporting electrolyte TBAP changed from 0.01, 0.1, to 0.2 M. As showed in Figure 6a–c, when the concentration of electrolyte gradually increased in the polymerization process, the surface of the modified electrode tended to be rougher. This result indicated that the excess of TBAP hindered self-assembly of PEM to a regular structure, reducing the reactive surface area. On the other hand, when the concentration of TBPA was too low (i.e., 0.01 M), PEM appeared to be an irregular tubular structure (Figure 6a). The irregular stacking of PEM limited MOF525 being adsorbed onto the tubular inner layer. In addition, electron transmission was not following one dimension in the case of PEM stacking, which could hamper the redox performance. The CV trances displayed a slight difference to TBAP variations in the synthesis conditions (Figure 6d), where the S/B ratio varied from 1.15 to 1.19 and maintained at a low level (Table 1). Therefore, altering electrolyte concentration alone was not effective to achieve the enhancement of S/B ratio in this study.

**Figure 7:**
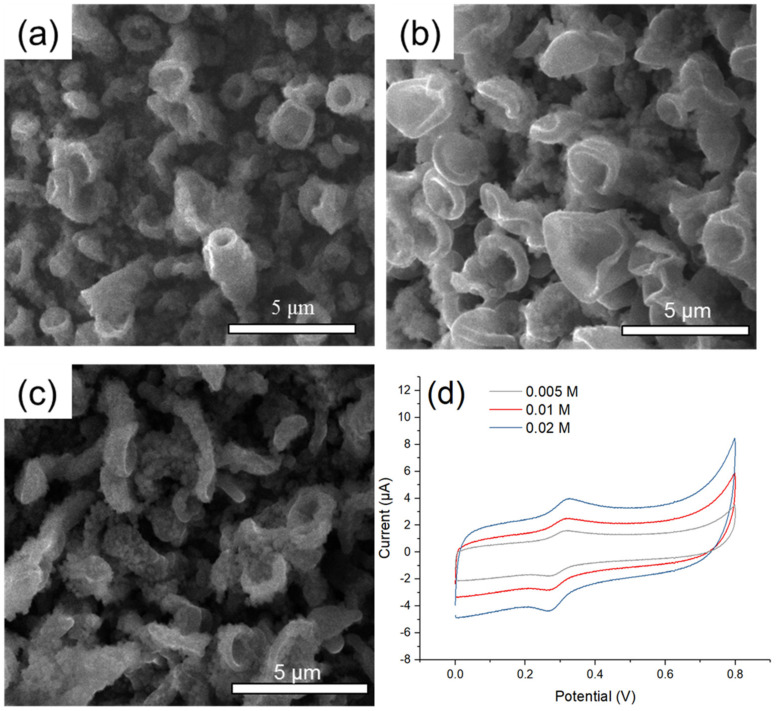
The SEM images of MOF525/PEM composites synthesized under: (**a**) 0.005 M; (**b**) 0.01 M; (**c**) 0.02 M TBAP solution at −42.5 °C; and (**d**) the CV traces of MOF525/PEM composites modified microelectrode synthesized under various TBAP concentrations at −42.5 °C.

Electropolymerization under a low temperature has been reported to facilitate formation of regular microstructure [46,47]. To provide low temperature environment for electropolymerization, dry ice was added to acetonitrile solution to prepare an ice bath of −42.5 °C. Figure 7a–c showed the SEM images of MOF525/PEM composites modified microelectrode synthesized under low concentrations of TBAP solutions. Under a low temperature and a low TBAP concentration, the as-synthesis PEM possessed moderate regular structure in the same direction. The CV diagrams showed relatively low background currents and high S/B ratios under these synthesis conditions (Figure 7d and Table 1). Considering the Ag/Ag^+^ reference electrode may freeze under a lower temperature, and a low TBAP concentration would result in a significant increase of solution resistance, the redox reaction cannot be carried out under these circumstances. Even though different conditions were tested for PEM electropolymerization, the as-synthesis materials did not achieve a satisfactory S/B ratio for DA detection. We therefore go to employ EDOT-Ph-COOH instead of EDOT-MeOH for polymerization.

### 3.4. MOF525/PEpC Composites Modified Microelectrode

**Figure 8:**
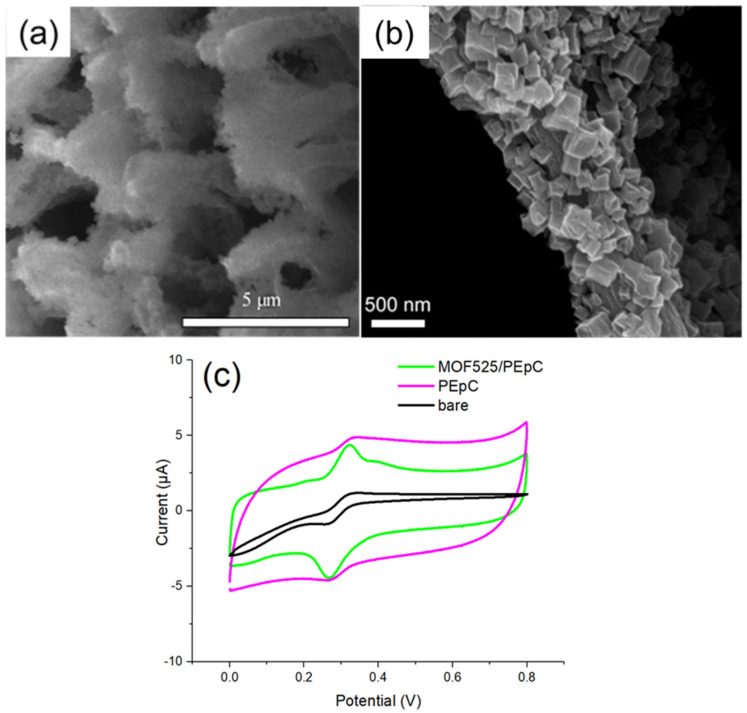
The SEM images of (**a**) MOF525/PEpC; (**b**) high-resolution MOF525/PEpC; and (**c**) the CV traces of unmodified and PEpC-based microelectrodes.

The functional groups in EDOT-Ph-COOH may facilitate the formation of regular structure under electropolymerization from various aspects. The strong hydrogen bond resulting from the carboxylic acid could better stabilize other molecules and form specific micelle structures (Prathish et al., 2014; Zhang et al., 2014). The benzene ring can provide a steric barrier between monomers and thus efficiently prevent the possible compacting or stacking polymerization [48,49]. Additionally, the π–π stacking from the benzene ring can also assist and stabilize micelles assembly [50]. The SEM image of the MOF525/PEpC showed a regular cylinder form with the diameter between 500 and 700 nm, which was much smaller than that with PEM (1–5 µm; Figure 8a). Therefore, PEpC can definitely provide a larger reactive surface than PEM. Under a unidirectional structure, the electron transfer can be more efficient and enhance sensor sensitivity. The high-resolution SEM confirmed that MOF525 was attached to the surface of PEpC (Figure 8b). The CV diagrams showed that the PEpC modified microelectrode was also catalytically active for DA (Figure 8c), where the catalytic capability may be ascribed to the carboxylic group [51]. The MOF525/PEpC modified microelectrode exhibited a more distinct oxidation peak, and the S/B ratio reached 1.74. The suitable potential window was between 0 and 0.7 V. Adjusting the electropolymerization time from 60 to 120 s could further optimize the S/B ratio (Table 2). It was found that when the electropolymerization time was 100 s, the S/B ratio was the highest as 2.02. Therefore, MOF525.PEpC composites synthesized by electropolymerization in 100 s were used in further study to modify the microelectrode for DA detection.

### 3.5. Detection of Dopamine by Differential Pulse Voltammetry

**Figure 9:**
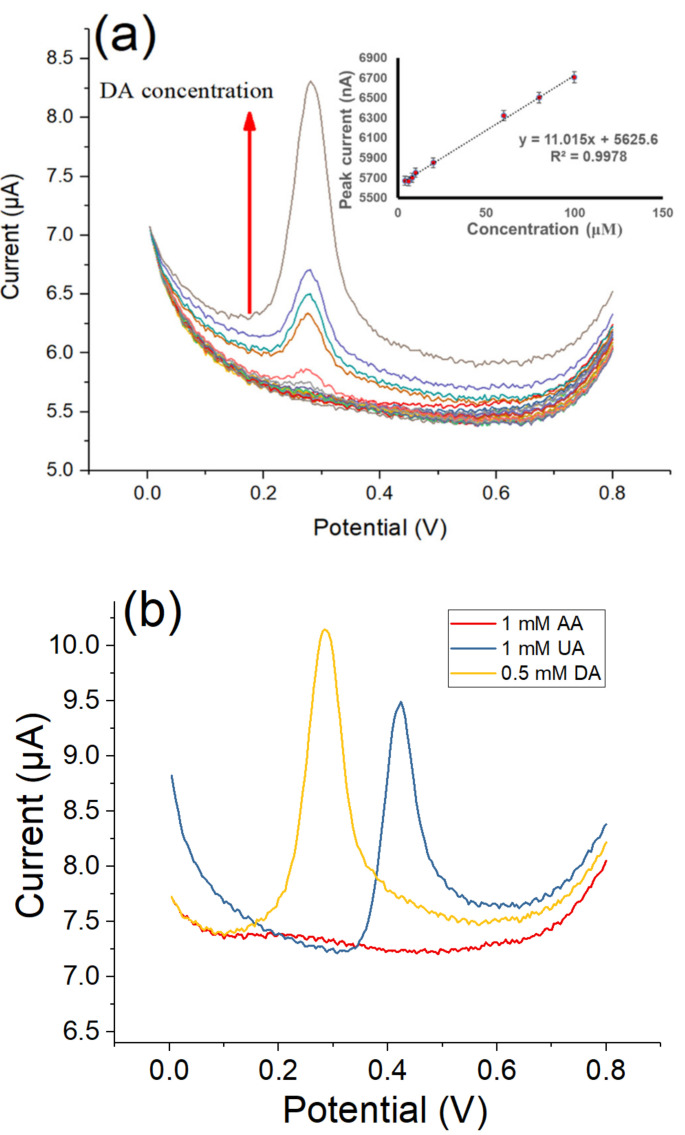

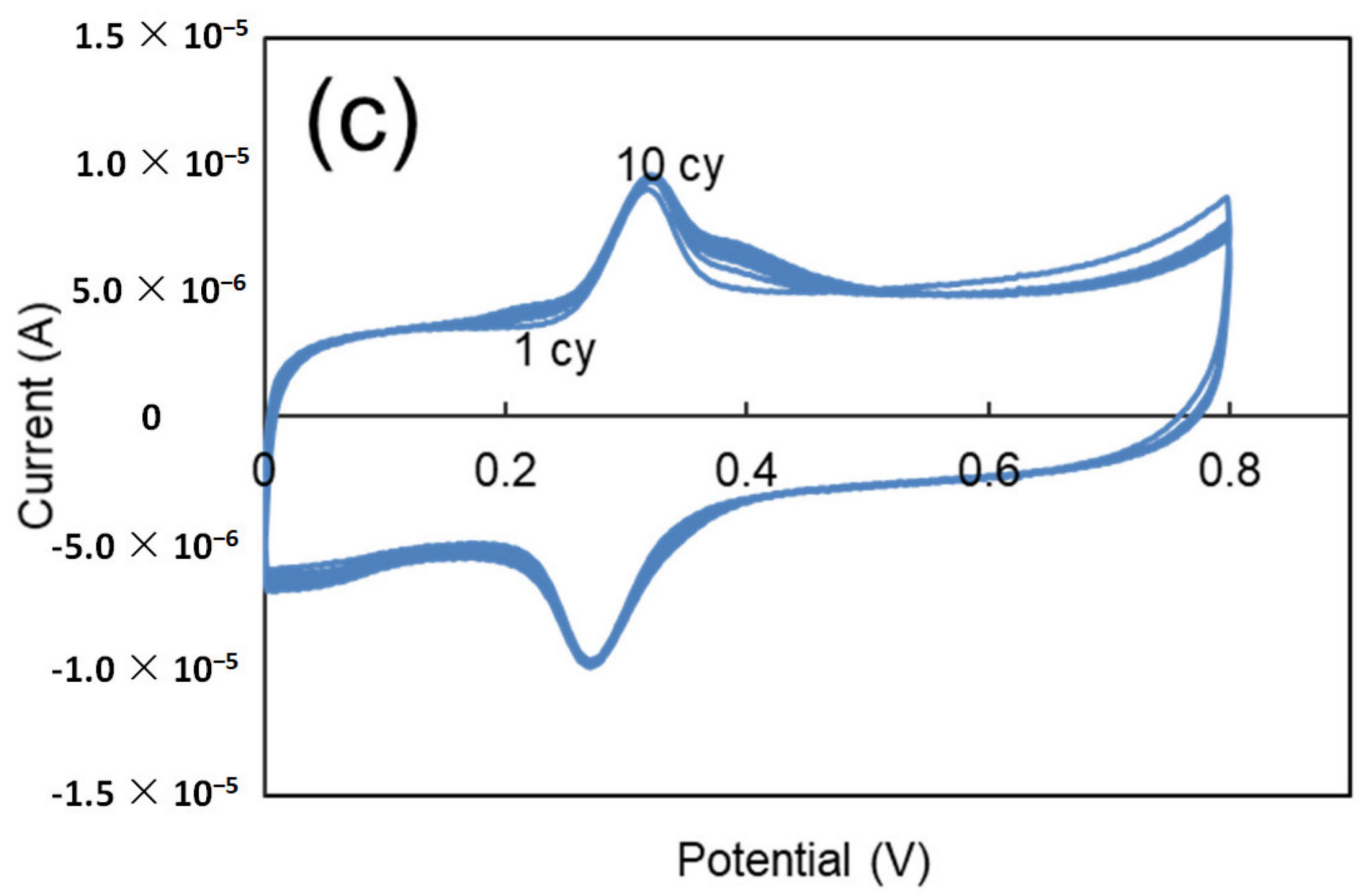
Differential pulse voltammetry (DPV) curves of (**a**) MOF525/PEpC modified microelectrode in ABS (pH = 5) with DA concentrations from 4 to 100 µM (Inset: linear dependence of the peak current against DA concentration), (**b**) detection of AA, UA and DA, respectively, and (**c**) stability of MOF525/PEpC modified microelectrodes in 10 cycles.

The electrochemical detection of DA on a microelectrode was further evaluated by the DPV technique. Figure 9a showed the DPV traces of the MOF525/PEpC modified microelectrode in ABS (pH = 5) with various DA concentrations. The catalytic peak current exhibited a linear increase with increasing DA concentration. The MOF525/PEpC modified microelectrode reached a sensitivity of 11 nA/μM in the range of the DA concentration between 4 and 100 µM (*R*^2^ = 0.998), which is comparable to the results of previous MOF525/PEDOT nanotube composite films [26]. Ascorbic acid (AA) and uric acid (UA) were also tested to study the interference of chemically similar analytes. In Figure 9b, there was no peak current of AA, and the peak current of UA was at 0.42 V, which did not overlap with the peak of DA at 0.28 V. In addition, the peak intensity of DA at 0.5 mM was larger than that of UA at 1 mM. These results confirmed that the MOF525/PEpC modified microelectrode exhibited high sensitivity and selectivity to DA detection. To examine the stability of MOF525/PEpC modified microelectrode, 10 continuous scan cycles were performed in ABS at a scan rate of 30 mV/s. As shown in Figure 9c, the background current exhibited a negligible decrease in ten cycles. The reproducibility of the microelectrode was also investigated by fabricated two other sensors under the same preparation protocol and tested in the same analyte solution. The as-synthesized microelectrodes showed excellent reproducibility.

Apart from PEpC enhancing S/B ratio, meso-tetra (4-carboxyphenyl)-porphyrin (TCPP) of MOF525 may also play a role in electrocatalysis and selectivity of DA. Under the electrochemical conditions of sensing, TCPP were readily protonated to TCPP^+^, which can associate with the primary amine group of DA and form stable ammonium ion [37,52]. Therefore, the sensitivity of MOF525 towards DA was ascribed to the interaction between the electron lone pair in DA and the radical cationic TCPP^+^. Meanwhile, the secondary amine group of UA was less attracted to TCPP^+^ because it readily formed an ammonium ion with H^+^ in the solution (Huang et al., 2017). Furthermore, the ammonium ion of UA was electrostatic repelling to TCPP^+^, which resulted in weaker current response than DA. Compared with the amine groups in DA and UA, the hydroxyl groups in AA exhibited a weak affinity to TCPP^+^, and therefore the current response was subtle (Eskelsen et al., 2012). In addition, the MOF525 nanocrystals also facilitated the diffusion of DA to the electrode surface, which led to a sensitive electrocatalytic response in the microelectrode detection. It is clear from this study that the surface morphology of PEDOT-based polymers had a significant influence on the sensitivity of DA detection by promoting the electron transmission, while MOF525 not only provided more reactive sites for electrocatalysis of DA but also enhanced the selectivity toward DA due to the presence of TCPP in the structure.

## 4. Conclusions

The MOF525 and PEDOT based polymer were synthesized as the composites to modify the microelectrode for DA detection. Two types of PEDOT were investigated in this study, and the results revealed that the carboxylic group in EDOT-Ph-COOH facilitated the formation of regular surface morphology under electropolymerization, which enhanced electron transmission efficiency in one dimension. The MOF 525 not only provided a large electrocatalytic surface but also promoted selectivity toward DA against AA and UA in view of the presence of porphyrin-based ligand in the MOF structure. The combination of MOF525 and PEDOT resulted in a much higher catalytic current than individual constituents, and the sensitivity reached 11 nA/μM with a good linear range of 4–100 µM. This study clearly demonstrated the great potential of MOF525/PEDOT composite modified microelectrode as a practical biosensor for future in vivo or in vitro analysis.

## Figures and Tables

**Table 1:** The S/B ratio of DA detection by MOF525/PEM modified microelectrode under various synthesis conditions.

Conditions		S/B Ratio
EDOT-MeOH:EDOT ^a^	3:1	1.32
	2:1	1.22
	1:1	1.21
	0.33:1	1.17
	0.2:1	1.11
TBAP concentration (M)	0.01	1.19
	0.1	1.17
	0.2	1.15
TBAP concentration (M) ^b^	0.005	1.70
	0.01	1.26
	0.02	1.26

^a^ Total concentration of monomers is 10 mM; ^b^ electropolymerization at −42.5 °C.

**Table 2:** Effect of PEpC polymerization time on the S/B ratio.

Polymerization Time (s)	S/B Ratio
60	1.68
80	1.74
100	2.02
120	1.39
